# Research progress on potential high-risk drugs for Stevens-Johnson syndrome and toxic epidermal necrolysis with insights from adverse drug reaction database mining

**DOI:** 10.3389/fphar.2026.1775888

**Published:** 2026-06-02

**Authors:** Houci Yang, Junjun Xu, Jiali Zhang, Xiaoyu Zhang, Chenghui Jin, Haibin Dai, Mingdong Yang

**Affiliations:** Department of Pharmacy, Second Affiliated Hospital, Zhejiang University School of Medicine, Hangzhou, Zhejiang, China

**Keywords:** adverse drug reaction (ADR), data mining, pharmacovigilance, Stevens-Johnson syndrome (SJS), toxic epidermal necrolysis (TEN), the FDA adverse event reporting system (FAERS), time to onset (TTO)

## Abstract

Stevens-Johnson Syndrome (SJS) and Toxic Epidermal Necrolysis (TEN) are rare yet life-threatening severe cutaneous adverse drug reactions (SCARs), characterized by high mortality and substantial morbidity risks. Identifying potential high-risk drugs associated with SJS/TEN is crucial for guiding clinical preventive interventions, enabling early detection, and enhancing risk management. With the rapid advancement of data science, adverse drug reaction (ADR) database mining has emerged as a powerful tool for systematically investigating the drug-SJS/TEN association, effectively overcoming the limitations of traditional case reports and small-sample studies regarding data scale and conclusion generalizability. This review summarizes recent advances in identifying potential high-risk drugs for SJS/TEN based on ADR database mining, with all included studies retrieved from peer-reviewed journals and strictly focused on SJS/TEN. We classify and discuss the major potential high-risk drug categories, including antibiotics, antiepileptics, allopurinol, nonsteroidal anti-inflammatory drugs (NSAIDs), proton pump inhibitors (PPIs), immune checkpoint inhibitors (ICIs), novel antiandrogens, carbonic anhydrase inhibitors, antivirals, and others. We also summarize their associated genetic susceptibilities, median onset times, and underlying mechanisms. These findings provide valuable references for enhancing medication safety and mitigating severe adverse drug reactions in clinical practice.

## 1 Introduction

Stevens-Johnson Syndrome (SJS) and Toxic Epidermal Necrolysis (TEN) are rare but life-threatening immune-mediated diseases. The initial manifestations of SJS/TEN are often nonspecific “flu-like” symptoms (e.g., general malaise, fever, anorexia, headache, rhinitis, cough, sore throat, and myalgia) in approximately one-third of cases (Dodiuk-Gad et al., 2015); alternatively, onset may involve mucosal involvement or an nonspecific rash. Cutaneous and mucosal (ocular, oral, and genital) inflammation, pain, and other systemic manifestations typically develop 4–28 days after the initiation of the culprit drug and progress rapidly (Dodiuk-Gad et al., 2015; Castellana et al., 2025). SJS/TEN rarely occurs beyond 8 weeks of starting the implicated medication (Lerch and Pichler, 2004). SJS affects less than 10% of the Body Surface Area (BSA), TEN involves more than 30%, and cases with 10%–30% epidermal involvement are categorized as the SJS-TEN overlap syndrome (Bastuji-Garin et al., 1993). These subtypes also differ significantly in fatality rates: SJS has a fatality rate of 2% (9/597), the SJS-TEN overlap syndrome 15% (26/171), and TEN the highest at 33% (74/223) (Traikia et al., 2020). Epidemiologically, SJS is more prevalent than TEN in clinical practice (Charlton et al., 2020). Compared with the general population, patients diagnosed with SJS/TEN face a substantial life expectancy loss of 9.43 years following diagnosis (Chiu and Chiu, 2023).

Given that 75.0%–89.7% of SJS/TEN cases are drug-induced (Mockenhaupt, 2011; Micheletti et al., 2018), accurately identifying potential high-risk culprit drugs has long been a core challenge in the prevention and clinical management of this condition. However, current clinical diagnosis and management of SJS/TEN are plagued by a dual dilemma. Early manifestations overlap extensively with common drug eruptions and infectious diseases, and the absence of specific diagnostic biomarkers leads to frequent misdiagnosis and delayed intervention. While the Algorithm of Drug Causality for Epidermal Necrolysis (ALDEN) is a widely used tool for determining drug causality in SJS/TEN, its underlying assumptions limit precision (Parvathaneni et al., 2025). Importantly, patients with a confirmed drug-related etiology exhibit a lower mortality rate than those with other underlying causes (Sekula et al., 2013). Conventional SJS/TEN research has relied heavily on case reports, cohort studies and systematic reviews. While these studies have confirmed classic causative drugs, their restricted scope makes it challenging to detect medication risks linked to rare and newly approved drugs, particularly amid the growing use of polypharmacy and the ongoing launch of new medications. In recent years, scholars have significantly supplemented traditional literature by mining SJS/TEN data from large-scale databases such as the FDA Adverse Event Reporting System (FAERS), the European Medicines Agency’s EudraVigilance, and the Japanese Adverse Drug Event Report Database (JADER). Therefore, it is urgent to integrate evidence from classic literature and data-mining studies to systematically to establish a comprehensive potential high-risk drug profile for SJS/TEN.

This review (Yang et al., 2025a) summarizes all SJS/TEN-associated potential high-risk medications and their onset times reported to date, aiming to provide robust theoretical support for the clinical prevention and management of SJS/TEN and further improve medication safety in routine clinical practice. For research articles (including reviews and database-mining studies), strictly SJS/TEN-relevant peer-reviewed journal publications as well as non-peer-reviewed medRxiv preprints (only one, Mukherjee et al., 2025a) were included to ensure the quality and thematic focus of this review. For case reports, formal inclusion and exclusion criteria were applied. Specifically, case reports were included if they were published in peer-reviewed journals, provided detailed clinical and diagnostic information consistent with a diagnosis of SJS/TEN, and contained complete individual patient data. Duplicate cases, narrative reviews lacking original case data, and studies with unavailable full text were excluded.

## 2 Comprehensive compilation of SJS/TEN-associated potential high-risk drugs

**FIGURE 1:**
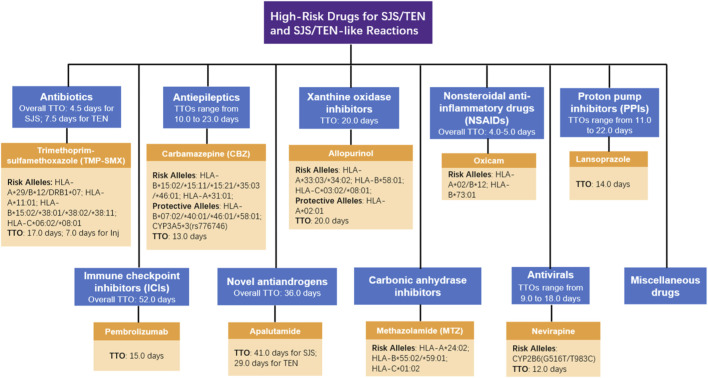
Drug classes, representative drugs, and key information implicated in SJS/TEN. Note: SJS/TEN = Stevens-Johnson syndrome/toxic epidermal necrolysis; HLA = human leukocyte antigen; TTO = median time to onset; CYP = Cytochrome P450; Inj = injection.

Potential high-risk drugs for SJS/TEN, such as lamotrigine (LTG), allopurinol, phenytoin, carbamazepine (CBZ), amoxicillin, trimethoprim-sulfamethoxazole (TMP-SMX), rifampin, levetiracetam, and vancomycin, have undergone label revisions through the FDA’s Drug Safety-related Labeling Changes (SrLC) program since 2016—reflecting regulatory efforts to mitigate these life-threatening cutaneous adverse reactions (Mukherjee et al., 2025a). A multicenter retrospective study conducted in the United States quantified the pharmaceutical etiologies of SJS/TEN: antibiotics accounted for 48.8% of cases (with TMP-SMX at 26.3%, β-lactam antibiotics at 12.4%, and fluoroquinolones at 3.6%), antiepileptics/mood stabilizers for 23.7% (phenytoin 9.5%, LTG 8.9%, CBZ 2.1%, phenobarbital 1.2%), allopurinol for 8.6%, and nonsteroidal anti-inflammatory drugs (NSAIDs) for 5.3% (Micheletti et al., 2018). These findings are corroborated by signal detection in international pharmacovigilance databases, including FAERS, which have yielded consistent patterns (Mukherjee et al., 2025a; Li et al., 2025). Notably, beyond these well-characterized culprit drugs, the proportion of SJS/TEN cases linked to the newly implicated agents, including immune checkpoint inhibitors (ICIs) and proton pump inhibitors (PPIs), has exhibited a steady upward trend in recent years, which underscores an evolving landscape of drug-induced SJS/TEN risk (Mukherjee et al., 2025a; Li et al., 2025; Fei et al., 2023). Below is a detailed breakdown of the key drug classes implicated in SJS/TEN, along with their etiological, genetic, mechanistic, and clinical profiles. These drug classes, representative drugs and their key information are presented in Figure 1.

### 2.1 Antibiotics

Infection is recognized as a risk factor for SJS/TEN (Imatoh and Saito, 2021). Notably, over half of drug-induced SJS/TEN cases are attributed to antibiotics (Yan et al., 2025; Blumenthal et al., 2015), though Micheletti RG et al.'s study reported a slightly lower proportion of 48.8% (Micheletti et al., 2018). Table 1 summarizes the antibiotic classes linked to SJS/TEN and the detailed list of implicated antibiotics.

**TABLE 1:** Antibiotics associated with SJS/TEN and relevant genetic associations.

Antibiotic classes	Medications	HLA
sulfonamides/synergists	sulfamethoxazole/trimethoprim (Micheletti et al., 2018; Yan et al., 2025) sulfasalazine (Imatoh and Saito, 2021), sulfadiazine (Imatoh and Saito, 2021), sulfadoxine (Imatoh and Saito, 2021), sulfafurazole (Imatoh and Saito, 2021)	HLA-A*29/B*12/DRB1*07 (Roujeau et al., 1987); HLA-A*11:01 (Sukasem et al., 2020); HLA-B*15:02 (Sukasem et al., 2020; Kongpan et al., 2015); HLA-B*38:01/*38:02/*38:11 (Lonjou et al., 2008); HLA-C*06:02 (Kongpan et al., 2015); HLA-C*08:01 (Sukasem et al., 2020; Kongpan et al., 2015; Nakkam et al., 2022)
penicillins	floxacillin (Frey et al., 2018), amoxicillin (Yan et al., 2025), piperacillin (Yan et al., 2025), phenoxymethylpenicillin (Frey et al., 2018), piperacillin/tazobactam (Fei et al., 2023)	​
cephalosporins	cefuroxime (Yan et al., 2025), ceftriaxone (Yan et al., 2025),cefotaxime (Yan et al., 2025),cephalexin (Frey et al., 2018), cephradine (Frey et al., 2018), cefadroxil (Frey et al., 2018),ceftazidime (Fei et al., 2023)	-
macrolides	azithromycin (Yan et al., 2025), erythromycin (Frey et al., 2018), clarithromycin (Frey et al., 2018)	-
aminoglycosides (Fukasawa et al., 2023)	gentamicin (Yan et al., 2025)	-
tetracyclines	doxycycline (Yan et al., 2025), lymecycline (Frey et al., 2018)	-
quinolones (Fukasawa et al., 2023)	ciprofloxacin (Frey et al., 2018), norfloxacin (Frey et al., 2018), levofloxacin (Wang et al., 2024)	HLA-B*15:02 (Wang et al., 2024)
nitroimidazoles	metronidazole (Frey et al., 2018; Chen et al., 2003)	-
oxazolidinones	linezolid (Ni et al., 2022; Li et al., 2024a)	-
lincomycins (Fukasawa et al., 2023)	lincomycin	-
glycopeptides (Fukasawa et al., 2023)	vancomycin (Yan et al., 2025; Ni et al., 2022; Li et al., 2024a)	-
fosfomycin (Fukasawa et al., 2023)	fosfomycin	-
carbapenems (Fukasawa et al., 2023)	-	-

SJS/TEN, Stevens-Johnson syndrome/toxic epidermal necrolysis; HLA, human leukocyte antigen; “-” = missing data due to no relevant literature reports.

#### 2.1.1 The highest-risk and newly identified antibiotics

Among antibiotic-associated SJS/TEN cases, sulfonamides are the most frequently implicated class (32%), followed by penicillins (22%), cephalosporins (11%), fluoroquinolones (4%), and macrolides (2%) (Lee et al., 2023). Sulfonamides also carry the highest absolute risk of SJS/TEN (Fukasawa et al., 2023). Within the sulfonamide class, TMP-SMX is most commonly identified as the causative agent (Micheletti et al., 2018; Yan et al., 2025), although other sulfonamide antibiotics including sulfadiazine, sulfadoxine, and sulfafurazole have also been reported to induce SJS/TEN (Imatoh and Saito, 2021). Human leukocyte antigen (HLA) gene associations with sulfonamide-induced SJS/TEN have been extensively investigated. Specific HLA alleles implicated include HLA-A*29/B*12/DRB1*07 (Roujeau et al., 1987), HLA-A*11:01 (Sukasem et al., 2020), HLA-B*15:02 (Sukasem et al., 2020; Kongpan et al., 2015), HLA-B*38:01/*38:02/*38:11 (Lonjou et al., 2008), HLA-C*06:02 (Kongpan et al., 2015), and HLA-C*08:01 (Sukasem et al., 2020; Kongpan et al., 2015; Nakkam et al., 2022), as summarized in Table 1. Notably, several of these alleles exhibit relatively high population frequencies in Han Chinese individuals (Shen et al., 2014): HLA-A*11:01 (26.8%), HLA-B*15:02 (3.8%–17.7%) (Wang et al., 2011a; Gonzalez-Galarza et al., 2020; Li et al., 2006), HLA-B*38:02 (3.1%), HLA-C*06:02 (5.0%), and HLA-C*08:01 (4.0%). While analyses of the JADER and FAERS databases demonstrated a strong association between infection and SJS/TEN in Western countries and Japan, such a correlation was not identified in Chinese populations (Imatoh and Saito, 2021). Nevertheless, owing to the high prevalence of the above-mentioned high-risk HLA alleles in the Chinese population, Chinese patients still require close monitoring for SJS/TEN, especially when receiving TMP-SMX therapy. Additionally, HLA-B*15:02 positivity was detected in one case of levofloxacin-induced SJS/TEN (30); however, a definitive causal relationship cannot be established due to the limited evidence from a single isolated case report. To date, no consistent HLA associations have been reported for other classes of antibiotics.

In a case-crossover study evaluating antibiotic-SJS/TEN associations using ALDEN with 170 participants, glycopeptides ranked second among antibiotic classes following TMP-SMX (Fukasawa et al., 2023). Several other antibiotic classes, including lincomycins, aminoglycosides, fosfomycins, and carbapenems, have been newly identified as potential risk factors for SJS/TEN (Fukasawa et al., 2023). These represent emerging, exploratory signals rather than well-established associations, and their clinical relevance remains preliminary and requires further validation.

#### 2.1.2 Infection site and age

A FAERS-based study conducted between Q1 2014 and Q4 2023, encompassing 1,221 cases of antibiotic-induced SJS/TEN, revealed that patients with pneumonia, urinary tract infections, or sepsis face a higher risk of developing SJS/TEN (Yan et al., 2025). The median age of patients with antibiotic-induced SJS was 63 years, while that of those with antibiotic-induced TEN was 60 years (Yan et al., 2025). In a case-crossover study of 170 SJS/TEN cases from Japanese insurance claims data (1 Jan 2005 to 31 Oct 2020) that excluded individuals aged ≥75 years, the mean age of patients with antibiotic-induced SJS/TEN was 45.1 years (SD 16.0) (Fukasawa et al., 2023). Collectively, these findings indicate that middle-aged and elderly individuals constitute the potential high-risk population for antibiotic-induced SJS/TEN. Notably, vancomycin and linezolid are frequently used in middle-aged patients (45–64 years) at risk of SJS/TEN (Ni et al., 2022).

#### 2.1.3 Gender

Male patients are reported to have an overall higher risk of antibiotic-induced SJS/TEN (Yan et al., 2025). This elevated risk is particularly evident in males receiving specific antibiotics, including amoxicillin, cefuroxime, doxycycline, piperacillin, and TMP-SMX, with no significant age-related differences noted in this association. However, the aforementioned Japanese study (Fukasawa et al., 2023) showed that females accounted for 62.4% of antibiotic-induced SJS/TEN cases in its cohort. This gender distribution discrepancy suggests a potentially lower susceptibility to antibiotic-induced SJS/TEN among female patients aged ≥75 years, a subgroup that was excluded from the Japanese study. Among cases limited to vancomycin-induced SJS alone, males account for a slightly higher proportion (49.28%) than females (43.84%) (Ni et al., 2022). However, female patients have been reported to carry an overall increased risk of vancomycin-associated SJS/TEN (17), suggesting that the more severe TEN subtype occurs more frequently in females relative to SJS. The gender distribution of linezolid-related SJS cases was equal between males and females (44.44% each) (Ni et al., 2022). Pharmacologically, vancomycin carries a higher risk of severe cutaneous adverse reactions (SCARs) compared with linezolid (Li et al., 2024a), and linezolid is also less prone to inducing SJS (Ni et al., 2022).

#### 2.1.4 Pediatric population

While research on SJS/TEN in children is less extensive than in adults, studies have demonstrated age-related differences in the risk of antibiotic-associated SJS/TEN. Specifically, systemic antibiotics such as amoxicillin, azithromycin, and cefaclor are more frequently implicated in young children aged 0–11 years (Bayram-Ozgur et al., 2025). By contrast, vancomycin and linezolid are less commonly associated with SJS/TEN in pediatric patients younger than 18 years (Ni et al., 2022).

#### 2.1.5 Non-systemic administration and SJS/TEN risk

Beyond systemic administration, a rare case of antibacterial eye drop-induced TEN was reported in Japan (Hashimoto et al., 2025). This case confirms that SJS/TEN is a severe, dose-independent hypersensitivity reaction, which can be triggered by trace amounts of eye drops in susceptible individuals. The reaction occurs via ocular mucosal absorption—specifically through the conjunctiva and nasolacrimal duct—allowing minimal drug entry into the systemic circulation. This finding underscores that eye drops are not absolutely systemically safe and emphasizes the importance of adhering to allergy warnings, particularly for potential high-risk drugs.

### 2.2 Antiepileptics

Antiepileptic drugs (AEDs) are among the primary medications associated with SJS/TEN. An analysis of the FAERS database, focusing on AED-induced SJS/TEN cases from July 2014 to December 2017, revealed that specific AEDs—including zonisamide, rufinamide, clorazepate, LTG, phenytoin, and CBZ—were associated with a 9-fold increased risk of SJS/TEN compared to non-AED medications (Borrelli et al., 2018). The incidence of AED-induced SJS/TEN varies across major global populations: the rate is significantly higher in Han Chinese (8 per million person-years) than in Caucasians (2–3 per million person-years) (Chung et al., 2004). Genetic factors, particularly the extensively studied HLA system, play a pivotal role in this susceptibility. AEDs implicated in SJS/TEN and their corresponding genetic associations are summarized in Table 2. Among antiepileptic drugs, sodium channel blockers have been most extensively investigated for genetic associations: zonisamide (Borrelli et al., 2018; Hosohata et al., 2019; Qian et al., 2025; Vivar et al., 2018) was linked to HLA-A*02:07 (Tham et al., 2024); CBZ (Borrelli et al., 2018) to HLA-A*31:01 and HLA-B*15:02/*15:11/*15:21//*35:03/*46:01; oxcarbazepine (OXC) (Tham et al., 2024) to HLA-B*15:02 (Chen et al., 2017; Yun et al., 2011); LTG (Borrelli et al., 2018; Hosohata et al., 2019; Qian et al., 2025) to HLA-B*15:02/*38:01/*38:11 (Lonjou et al., 2008) and HLA-DRB1*04:05/DQB1*04:01/DQA1*03:03 (Ito et al., 2015); phenytoin (Borrelli et al., 2018; Hosohata et al., 2019) to HLA-B*15:02/*51:01 (Kaniwa et al., 2013), HLA-B*13:01/C*08:01/DRB1*16:02 (Hung et al., 2010), and CYP2C9 (Depondt et al., 2011). Among GABAergic-enhancing agents, phenobarbital (Hosohata et al., 2019) was associated with HLA-B*51:01 (Kaniwa et al., 2013).

**TABLE 2:** Antiepileptics associated with SJS/TEN and relevant genetic associations.

Antiepileptic classes	Medications	HLA & CYP
sodium channel blockers	zonisamide (Borrelli et al., 2018; Hosohata et al., 2019; Qian et al., 2025; Vivar et al., 2018)	HLA-A*02:07 (Kaniwa et al., 2013)
	carbamazepine (Borrelli et al., 2018)	HLA-A*31:01; HLA-B*15:02/*15:11/*15:21/*35:03/*46:01
	oxcarbazepine (Tham et al., 2024)	HLA-B*15:02 (Chen et al., 2017; Yun et al., 2011)
	lamotrigine (Borrelli et al., 2018; Hosohata et al., 2019; Qian et al., 2025)	HLA-B*15:02; HLA-B*38:01/*38:11 (Lonjou et al., 2008) HLA-DRB1*04:05/DQB1*04:01/DQA1*03:03 (Ito et al., 2015)
	phenytoin (Borrelli et al., 2018; Hosohata et al., 2019)	HLA-B*15:02; HLA-B*51:01 (Kaniwa et al., 2013) HLA-B*13:01/C*08:01/DRB1*16:02 (Hung et al., 2010); CYP2C9 (Depondt et al., 2011)
	rufinamide(1 report) (Borrelli et al., 2018)	-
	eslicarbazepine (1 report) (Borrelli et al., 2018)	-
enhancing GABAergic system function	clobazam (Qian et al., 2025)	-
	clorazepate (1 report) (Borrelli et al., 2018)	-
	phenobarbital (Hosohata et al., 2019)	HLA-B*51:01 (Kaniwa et al., 2013)
	clonazepam (Hosohata et al., 2019)	-
	diazepam (Hosohata et al., 2019)	-
	lorazepam (1 report) (Borrelli et al., 2018)	-
multitarget mechanisms	valproic Acid (Hosohata et al., 2019)	-
	divalproex (Blumenthal et al., 2015) (≤3 reports) (Borrelli et al., 2018)	-
	topiramate (Blumenthal et al., 2015)	-
glutamate receptor antagonists/modulators	levetiracetam (Hosohata et al., 2019; He et al., 2024)	-
calcium channel blockers	ethosuximide (Hosohata et al., 2019)	-
	pregabalin (Borrelli et al., 2018)	-
	gabapentin (Blumenthal et al., 2015; Hosohata et al., 2019)	-
voltage-gated sodium channel slow inactivation enhancer + SV2A binder	lacosamide(1 report) (Borrelli et al., 2018)	-

SJS/TEN, Stevens-Johnson syndrome/toxic epidermal necrolysis; HLA, human leukocyte antigen; CYP, Cytochrome P450; SV2A = Synaptic Vesicle Glycoprotein 2A; “-“ = missing data due to no relevant literature reports.

#### 2.2.1 CBZ-related HLA and other genetic markers in SJS/TEN

##### 2.2.1.1 Risk alleles

Table 3 presents the most well-studied HLA alleles associated with CBZ-induced SJS/TEN, along with the allele frequencies of these alleles across different populations, SJS/TEN patients, SCARs patients, CBZ-tolerant individuals, and the general population. To date, HLA-B*15:02 demonstrates the strongest association with CBZ-induced SJS/TEN among all identified HLA genetic markers (Chung et al., 2004). High carrier frequencies of HLA-B*15:02 have been reported in Han Chinese populations (Wang et al., 2011a; Chung et al., 2004; Man et al., 2007), including those from Hong Kong (Man et al., 2007; Cheung et al., 2013) and Taiwan (Chung et al., 2004; Genin et al., 2014), as well as in Singaporean (Chong et al., 2014), Vietnamese (Nguyen et al., 2015), Thai (Tassaneeyakul et al., 2010), Malaysian (Chang et al., 2011), and Indian (Mehta et al., 2009) populations. Among these populations, allele frequencies reach 69.6%–100.0% in SJS/TEN patients, 3.0%–24.0% in CBZ-tolerant individuals, and 3.8%–17.7% in the general population. For detailed data, see Table 3. In contrast, HLA-B*15:02 is far less prevalent in Koreans (Kim et al., 2011), with frequencies of 14.3% in SJS/TEN patients, 4.2% in other SCARs patients, and 0.4% in the general population. Notably, this allele is absent in SJS/TEN patients of European and Japanese ancestry (Tangamornsuksan et al., 2013), with a general population frequency of only 0.05%–0.06% in Europeans (Ramírez et al., 2017).

**TABLE 3:** Distribution of key HLA alleles across carbamazepine-related populations: SJS/TEN, SCARs, tolerant individuals, and general population.

HLA	Population	SJS/TEN	SCARs	Tolerant	General
HLA-B*15:02	Han Chinese (Wang et al., 2011a; Shi et al., 2017; Shi et al., 2012; Zhang et al., 2011)	69.6% (39/56)-100.0% (9/9)	-	9.5% (2/21)-15.6% (28/179)	3.8%–17.7% (Wang et al., 2011a; Gonzalez-Galarza et al., 2020; Li et al., 2006)
	Han in Taiwan (Chung et al., 2004; Genin et al., 2014)	77.4% (41/53)-100.0% (44/44)	-	3.0% (3/101)-5.6% (4/72)	8.5% (60/710)-8.6% (8/93)
	Han in Hongkong (Man et al., 2007; Cheung et al., 2013)	92.3% (24/26)-100.0% (4/4)	75.0% (6/8)	11.9% (16/135)-14.5% (7/48)	17.9% (Gonzalez-Galarza et al., 2020)
	Singaporean (Chong et al., 2014)	100.0% (5/5)	-	10.0% (1/10)	-
	Vietnamese (Nguyen et al., 2015)	91.4% (32/35)	-	24.0% (6/25)	13.5% (Hoa et al., 2008)
	Thai (Tassaneeyakul et al., 2010)	88.1% (37/42)	-	11.9% (15/42)	16.1% (Gonzalez-Galarza et al., 2020)
	Malaysian (Chang et al., 2011)	75.0% (12/16)	-	-	15.7% (47/300)
	Indian (Mehta et al., 2009)	75.0% (6/8)	-	-	-
	Korean (Kim et al., 2011)	14.3% (1/7)	4.2% (1/24)	-	0.4% (2/485)
	European Caucasian (Wang et al., 2011a; Chung et al., 2004; Genin et al., 2014; Chung and Hung, 2012)	0.0% (0/20)	-	0.0% (0/43)	0.05% (4/8862)-0.06% (Ramírez et al., 2017)
	Japanese (Kaniwa et al., 2010)	0.0% (0/14)	-	-	-
HLA-B*15:11	Japanese (Kaniwa et al., 2010)	28.6% (4/14)	-	-	-
	Korean (Kim et al., 2011)	42.9% (3/7)	-	4.0% (2/50)	3.9% (19/485)
	Asian (Biswas et al., 2022)	-	-	-	∼2%
	Southern Han Chinese (Shi et al., 2017; Shi et al., 2012)	7.1% (4/56)-11.1% (2/18)	-	0.0% (0/179)	0.4% (1/264)
	European (Kloypan et al., 2021; Biswas et al., 2022)	-	-	-	0.0%
HLA-B*15:21	European Caucasians (Kloypan et al., 2021; Biswas et al., 2022)	-	-	-	0%–0.0003%
	Chinese (Biswas et al., 2022)	-	-	-	0.01%
	Vietnamese	-	-	-	0.3% (Hoa et al., 2008)
	Asian (Biswas et al., 2022)	-	-	-	0.03%–∼3%
	African (Biswas et al., 2022)	-	-	-	0.0%
	East Asian (Biswas et al., 2022)	-	-	-	0.0%
HLA-A*31:01	European (Genin et al., 2014; McCormack et al., 2011)	15.0% (3/20)	-	3.9% (10/257)	∼2–∼5% (McCormack et al., 2011), 4.5% (396/8862)
	Japanese (Ozeki et al., 2011)	83.3% (5/6)	60.7% (37/61)	12.5% (47/376)	∼9% (McCormack et al., 2011)-16.1% (Gonzalez-Galarza et al., 2020)
	Korean (Kim et al., 2011)	42.9% (3/7)	54.2% (13/24)	-	10.3% (50/485)
	Southern Han Chinese (Shi et al., 2012)	6.3% (1/16)	-	0.0% (0/32)	5.7% (15/264)
	Han in Taiwan (Genin et al., 2014)	1.9% (1/53)	-	4.2% (3/72)	3.7% (26/710)
	Vietnamese	-	-	-	2.1% (Hoa et al., 2008)

SJS/TEN, Stevens-Johnson syndrome/toxic epidermal necrolysis; HLA, human leukocyte antigen; SCARs: severe cutaneous adverse reactions; Tolerant = tolerant population exposed to the relevant trigger but without SJS/TEN, onset; General = unselected general population; “-” = missing data due to no relevant literature reports.

HLA-B*15:11 represents the predominant risk allele for CBZ-induced SJS/TEN in Japanese and Korean populations. It is detected in 28.6% and 42.9% of SJS/TEN patients in Japan (Kaniwa et al., 2010) and Korea (Kim et al., 2011), respectively, but in only 7.1%–11.1% of Han Chinese SJS/TEN patients (Shi et al., 2017; Shi et al., 2012). In the general population, the frequency is 3.9% in Koreans and merely 0.4% in Han Chinese, and the frequency of this allele is 0.0% in the European population (Kloypan et al., 2021; Biswas et al., 2022).

HLA-B*15:21, although rare in most populations, is also implicated in CBZ-induced SJS/TEN. This allele is found in Asian populations (allele frequency: 0.03%–3.0%) (Biswas et al., 2022). Specifically, its frequency is 0.3% in Vietnamese individuals (Hoa et al., 2008) and an extremely low rate of 0.01% in the overall Han Chinese population (Biswas et al., 2022). HLA-B*15:21 is nearly absent (0%–0.0003%) in European and African populations (Kloypan et al., 2021; Biswas et al., 2022). Previous studies have identified HLA-B*15:21 in a Thai SJS/TEN patient negative for HLA-B*15:02.

HLA-A*31:01 has been identified as the primary genetic determinant of CBZ-induced SJS/TEN in European, Japanese, and South Korean populations. The highest population frequencies are observed in Japan (Ozeki et al., 2011) (9.0% (McCormack et al., 2011)–16.1% (Gonzalez-Galarza et al., 2020)) and Korea (10.3%) (Kim et al., 2011), followed by southern Han Chinese (5.7%) (Shi et al., 2012), Europeans (2.0%–5.0%) (McCormack et al., 2011), Taiwanese Han Chinese (3.7%) (Genin et al., 2014), and Vietnamese (2.1%) (Hoa et al., 2008). Among SJS/TEN patients, HLA-B*31:01 frequencies are markedly highest in Japanese patients (83.3%) (Ozeki et al., 2011), followed by Koreans (42.9%) (Kim et al., 2011), Europeans (15.0%) (Genin et al., 2014; McCormack et al., 2011), southern Han Chinese (6.3%) (Shi et al., 2012), and Taiwanese Han Chinese (1.9%) (Genin et al., 2014). Importantly, HLA-A*31:01 has consistently been associated with an increased risk of CBZ-induced SCARs across diverse ethnic groups (Amstutz et al., 2014), and its genetic testing is recommended in the drug labels in some countries (Manson et al., 2020).

In China, CBZ-induced SJS patients negative for HLA-B*15:02 have been found to carry alternative risk alleles, including HLA-A*24:02 (Shi et al., 2017; Shi et al., 2012), HLA-B*15:11 (Sun et al., 2014), HLA-B*35:03 (Zhang et al., 2011), and HLA-B*46:01 (Zhang et al., 2011). Beyond the HLA system, polymorphisms in CYP2C19*2 (681G>A, rs4244285) and CYP2C19*3 (636G>A, rs4986893) are associated with increased susceptibility to CBZ-related SJS/TEN (Laska et al., 2017). In addition, the EPHX1 c.337T>C polymorphism may enhance the severity of SJS/TEN by increasing the concentration of CBZ-10,11-epoxide—a toxic metabolite of CBZ—in epilepsy patients (He et al., 2014).

##### 2.2.1.2 Protective alleles

HLA-B*07:02 acts as a potential protective genetic marker for severe CBZ hypersensitivity in Caucasians: this allele is entirely absent in patients with severe hypersensitivity yet present in CBZ-tolerant individuals and those with mild reactions, and even a single copy can markedly reduce the risk of severe hypersensitivity (Alfirevic et al., 2006). Its protective mechanism is linked to immune polarization, as it induces Th2 immune responses and counteracts the Th1-mediated pathogenesis of CBZ hypersensitivity, though further validation in independent cohorts is needed to confirm its clinical relevance. In the Asian population, HLA-B*40:01, HLA-B*46:01 and HLA-B*58:01 have been identified as strong protective factors against CBZ-induced SJS/TEN (Wang et al., 2017). Additionally, CYP3A5*3 is the most prevalent allele associated with diminished CYP3A5 enzymatic activity, and the absence of this variant (rs776746) has been proposed to confer a protective effect against CBZ-related hypersensitivity reactions in Brazilian individuals (Tanno et al., 2015).

#### 2.2.2 Risk of cross-reactivity among AEDs for SJS/TEN

Compared with CBZ-induced SJS/TEN, OXC-associated SJS/TEN exhibits a lower incidence and milder severity (Chen et al., 2017), while LTG- and phenytoin-induced SJS/TEN shows a weaker association with HLA-B*15:02 (Tham et al., 2024). A significant risk of cross-reactivity exists among AEDs, including CBZ, OXC, LTG, and phenytoin, with LTG being less frequently implicated (Bloch et al., 2014; Wang et al., 2010; Alvestad et al., 2008). However, a study analyzing the JADER database identified LTG as the AED with the strongest signal for drug-induced rashes (Hosohata et al., 2019). Many AEDs containing an aromatic ring in their chemical structure (e.g., CBZ, phenytoin, phenobarbital, zonisamide) are known to induce adverse cutaneous drug reactions through the formation of arene oxide intermediates (Knowles et al., 1999). These reactive metabolites bind to cellular macromolecules, leading to cell necrosis or secondary immunological responses (Wang et al., 2011b). In contrast, despite containing an aromatic ring, LTG does not generate such reactive metabolites (Lu and Uetrecht, 2007); instead, it induces adverse cutaneous reactions via an allergen-mediated pathway (Naisbitt et al., 2003), although its precise mechanism remains unclear. Caution is therefore advised when prescribing these AEDs, particularly when switching from one agent to another.

#### 2.2.3 HLA-B*15:02 pretreatment screening and cost-effectiveness

Pretreatment screening for HLA-B*15:02 is recommended before initiating CBZ therapy in Asian populations, and national pre-CBZ HLA-B*15:02 screening guidelines have been established in countries such as Thailand (Kloypan et al., 2021). Pharmacogenomic testing for HLA-B*15:02 has been shown to be cost-effective in populations where the allele prevalence exceeds 5% or is at least 2.5% (Kloypan et al., 2021; Nguyen et al., 2019; Dong et al., 2012). Even in the absence of universal subsidy for HLA-B*15:02 screening, it remains more cost-effective than no screening for Asian Australian epilepsy patients (Gu et al., 2024). Carriers of HLA-B*15:02 should avoid aromatic AEDs (e.g., CBZ, OXC, phenytoin) and use LTG with caution (Hung et al., 2010). The implementation of HLA-B*15:02 screening has led to significant reductions in CBZ prescriptions: from 16.2% to 2.6% in Hong Kong post-2008 (Chen et al., 2014), and from 6% to 4% in Taiwan following the official reimbursement of pre-CBZ HLA-B*15:02 genotyping in June 2010 (Chang et al., 2023). Other potential risk alleles (e.g., HLA-B*15:11, HLA-B*15:21, and HLA-A*31:01) are not uniformly included in global clinical guidelines or drug labeling (Kloypan et al., 2021).

#### 2.2.4 Newer AEDs associated with SJS/TEN

In addition to well-characterized SCARs-inducing AEDs such as CBZ and LTG, valproic acid (Hosohata et al., 2019) and levetiracetam (Hosohata et al., 2019; He et al., 2024) have emerged as significant contributors to these adverse reactions (Park et al., 2019). A case of levetiracetam-induced SJS has been reported in a child (Zou et al., 2012). Among pediatric populations, AED-associated SJS/TEN predominantly affects older children (12–17 years of age) (Bayram-Ozgur et al., 2025). Patients with a sulfonamide allergy must avoid zonisamide, and caution is also advised for individuals with no personal history of sulfonamide allergy but whose parents have such an allergy, as zonisamide may induce SJS/TEN in these cases (Majeres and Suppes, 2004). A Japanese study suggested that HLA-A*02:07 may serve as a susceptibility biomarker for zonisamide-induced SJS/TEN (Kaniwa et al., 2013). Genetic testing is recommended when conditions permit. Incorporating genetic testing into the routine prescribing of high-risk AEDs can normalize its utilization and substantially reduce the potential risk of SJS/TEN (de Olazarra and Wang, 2023). Other AEDs occasionally reported include the sodium channel blockers rufinamide and eslicarbazepine (Borrelli et al., 2018); GABAergic-enhancing agents clobazam (Qian et al., 2025), clorazepate (Borrelli et al., 2018), clonazepam (Hosohata et al., 2019), diazepam (Hosohata et al., 2019), and lorazepam (Borrelli et al., 2018); multitarget agents divalproex (Blumenthal et al., 2015; Borrelli et al., 2018) and topiramate (Blumenthal et al., 2015); calcium channel blockers ethosuximide (Hosohata et al., 2019), pregabalin (Borrelli et al., 2018), and gabapentin (Blumenthal et al., 2015; Hosohata et al., 2019); and the combined agent lacosamide (Borrelli et al., 2018). All AEDs associated with SJS/TEN are listed in Table 2.

### 2.3 Xanthine oxidase inhibitors

As reported in the EuroSCAR study, allopurinol—an extensively used xanthine oxidase inhibitor for hyperuricemia and gout management—was the leading cause of SJS/TEN in Europe and Israel (Halevy et al., 2008). Notably, allopurinol also ranks among the primary inducers of SJS/TEN in Asian populations (Lin et al., 2005). Various risk factors contribute to this severe adverse reaction, which are summarized below.

#### 2.3.1 Allopurinol dosage and risk of allopurinol-induced SJS/TEN

The risk of allopurinol-induced SJS/TEN is restricted to short-term use (≤8 weeks) and exhibits a dose-dependent pattern: daily doses of 200 mg or higher correlate with a significantly elevated risk (Halevy et al., 2008). Adherence to established allopurinol prescribing guidelines (Smith et al., 2000) may therefore substantially reduce the associated morbidity and mortality. Further support for a potential gene-dose effect comes from a study demonstrating that HLA-B*58:01 homozygosity confers an exceptionally high risk of developing this condition (Campbell et al., 2025).

#### 2.3.2 The role of HLA-B*58:01 in allopurinol-induced SJS/TEN

Multiple studies have validated a strong association between genetic predisposition and allopurinol-induced SCARs, including SJS/TEN, in Han Chinese populations, with HLA-B*58:01 identified as the major genetic risk locus (Hung et al., 2005; Cao et al., 2012). The allele frequency of HLA-B*58:01 is 14.0%–20.4% in the general Han Chinese population. Notably, 100% of Han Chinese patients with allopurinol-induced SJS/TEN and SCARs carry at least one copy of HLA-B*58:01, whereas this allele is present in only 11.1%–14.8% of allopurinol-tolerant Han individuals (Hung et al., 2005; Cao et al., 2012). Consistent evidence has been reported in Han Chinese populations from Taiwan and Hong Kong. In Taiwanese Han individuals, the general population frequency of HLA-B*58:01 is 20.0%, with carrier rates reaching 89.1%–92.3% in allopurinol-induced SJS/TEN patients and 17.4%–17.9% in tolerant subjects (Gonzalez-Galarza et al., 2020; Ng et al., 2016; Chung et al., 2015). In Hong Kong, 100.0% of patients with allopurinol-induced SCARs carry HLA-B*58:01, while only 13.3% of tolerant individuals test positive for this allele (Chiu et al., 2012). These findings collectively demonstrate that HLA-B*58:01 is a necessary but not sufficient condition for the development of allopurinol-induced SCARs/SJS/TEN.

A similar conclusion is supported by data from Thailand: the general population HLA-B*58:01 frequency is 10.1% in Thailand, with 100% of allopurinol-induced SJS/TEN patients and 96.7% of SCARs patients carrying this allele, compared to 4.0%–13.0% of tolerant controls (Tassaneeyakul et al., 2009; Sukasem et al., 2016). Similarly, in South Korea, the general population HLA-B*58:01 frequency is 12.2%, with carrier rates of 80.0% in allopurinol-induced SJS/TEN patients, 92.3% in SCARs patients, and 10.5% in tolerant individuals (Kang et al., 2011), highlighting a uniformly strong association across East Asian cohorts.

In stark contrast, the association between HLA-B*58:01 and allopurinol-induced SJS/TEN is considerably weaker in European and Japanese populations. Among Europeans, carrier rates in affected patients are 66.7% in Portugal (Gonçalo et al., 2013), 61.3% across Europe overall (55.6% for European ancestry) (Lonjou et al., 2008), and 20.0% in Italy (Cristallo et al., 2011). Similarly, positive rates in Japanese patients range from 40.0% to 55.6% (Tohkin et al., 2013; Kaniwa et al., 2008). Notably, general population frequencies of HLA-B*58:01 are substantially lower in both groups: 1.2% in Japanese individuals (Kaniwa et al., 2008; Tanaka et al., 1996), 2.0% in Portugal (Gonçalo et al., 2013), 1.5% in Europe overall (Lonjou et al., 2008), and 5.2% in Italy (Cristallo et al., 2011). With regard to SCARs specifically, HLA-B*58:01 carrier rates are 62.5% in US patients and 64.0% in Portuguese patients, corresponding to general population frequencies of 5.6% and 4.3%, respectively (Campbell et al., 2025; Gonçalo et al., 2013).

Collectively, these data reveal pronounced ethnic heterogeneity in the HLA-B*58:01-allopurinol SCARs association. East Asian populations (Han Chinese, Korean, and Thai) represent high-risk groups, making HLA-B*58:01 a clinically valuable pre-emptive screening biomarker in this cohort; conversely, the low baseline allele frequency and attenuated risk association limit its screening utility in European populations, warranting ethnicity-stratified strategies for allopurinol safety monitoring. All summarized data are presented in Table 4.

**TABLE 4:** Distribution of key HLA alleles across allopurinol-related populations: SJS/TEN, SCARs, tolerant individuals, and general population.

HLA	Population	SJS/TEN	SCARs	Tolerant	General
HLA-B*58:01	Han Chinese (Hung et al., 2005; Cao et al., 2012)	100.0% (13/13)	100.0% (38/38,51/51)	11.1% (7/63)-14.8% (20/135)	14.0% (80/572)-20.4% (19/93)
	Han in Taiwan (Gonzalez-Galarza et al., 2020; Ng et al., 2016; Chung et al., 2015)	89.1% (41/46)-92.3% (24/26)	90.6% (96/106)-96% (46/48)	17.4% (24/138)-17.9% (51/285)	20.0%
	Han in Hongkong (Chiu et al., 2012)	-	100.0% (19/19)	13.3% (4/30)	-
	Thai (Tassaneeyakul et al., 2009; Sukasem et al., 2016)	100.0% (27/27)	96.7% (29/30)	4.0% (4/100)-13.0% (7/54)	10.1% (111/1095)
	Korean (Kang et al., 2011)	80.0% (4/5)	92.3% (24/26)	10.5% (6/57)	12.2% (59/485)
	Japanese	40.0% (4/10) (Kaniwa et al., 2008)- 55.6% (10/18) (Tohkin et al., 2013)	-	-	1.2% (6/493) (Kaniwa et al., 2008; Tanaka et al., 1996)
	Portuguese (Gonçalo et al., 2013)	66.7% (4/6)	64.0% (16/25)	4.3% (1/23)	2.0% (63/3,200)
	European (Lonjou et al., 2008)	European population 61.3% (19/31) European ancestry 55.6% (15/27)	-	-	1.5% (28/1,822)
	American (Campbell et al., 2025)	-	62.5% (10/16)	5.6% (9/160)	2.3% (2162/94,489)
	Italian (Cristallo et al., 2011)	20.0% (4/20)	42.8% (3/7)	-	5.2% (6/115)
HLA-A*34:02	American (Campbell et al., 2025)	-	18.8% (3/16)	4.4% (7/160)	1.3% (1,246/94,489)
HLA-C*03:02	Korean (Kang et al., 2011)	80.0% (4/5)	92.3% (24/26)	12.3% (7/57)	20.4% (99/485)
	Japanese (Tohkin et al., 2013)	55.6% (10/18)	-	-	0.0% (0/117) (Tanaka et al., 1996)
	Italian (Cristallo et al., 2011)	10.0% (2/10)	28.6% (2/7)	-	0.0% (0/115)
HLA-A*33:03	Korean (Kang et al., 2011)	80.0% (4/5)	84.6% (22/26)	26.3% (15/57)	28.9% (140/485)
	Japanese (Tohkin et al., 2013)	44.4% (8/18)	-	-	15.8% (78/493) (Tanaka et al., 1996)
	Italian (Cristallo et al., 2011)	10.0% (2/10)	28.6% (2/7)	-	0.0% (0/115)
HLA-C*08:01	Italian (Cristallo et al., 2011)	10.0% (2/10)	28.6% (2/7)	-	0.0% (0/115)
HLA-A*02:01	Korean (Kang et al., 2011)	0.0% (0/26)	0.0% (0/5)	29.8% (17/57)	29.9% (145/485)

SJS/TEN, Stevens-Johnson syndrome/toxic epidermal necrolysis; HLA, human leukocyte antigen; SCARs, severe cutaneous adverse reactions; Tolerant = tolerant population exposed to the relevant trigger but without SJS/TEN, onset; General = unselected general population; “-” = missing data due to no relevant literature reports.

#### 2.3.3 Non-B*58:01 HLA alleles: risk and protective factors for allopurinol-induced SJS/TEN and SCARs

##### 2.3.3.1 Risk alleles

Beyond HLA-B*58:01, multiple additional HLA alleles have been identified as independent or supplementary genetic modifiers of allopurinol-induced SCARs/SJS/TEN across diverse populations. Notably, in a previously reported Portuguese cohort, the HLA-A*34 allele group (encompassing HLA-A*34:02) was detected in 5 of 9 HLA-B*58:01-negative patients with allopurinol-induced SCARs (Gonçalo et al., 2013). Subsequent US-based research further validated HLA-A*34:02 as a second independent genetic risk factor, with an allele frequency of 18.8% in SCARs patients, 4.4% in tolerant individuals, and 1.3% in the general population (Campbell et al., 2025).

In East Asian populations beyond Han Chinese, distinct HLA alleles confer heightened risk: in South Korean patients (Kang et al., 2011), HLA-C*03:02 was present in 80.0% of those with allopurinol-induced SJS/TEN and 92.3% of those with SCARs, compared to 12.3% in tolerant subjects and 20.4% in the general population; meanwhile, HLA-A*33:03 carrier rates reached 80.0% in SJS/TEN patients and 84.6% in SCARs patients, versus 26.3% in tolerant individuals and 28.9% in the general population. Parallel findings emerged in Japanese cohorts (Tohkin et al., 2013), where HLA-C*03:02 was detected in 55.6% of allopurinol-induced SJS/TEN patients (0.0% in the general population) and HLA-A*33:03 in 44.4% of affected patients (15.8% in the general population).

Italian research (Cristallo et al., 2011) further corroborated these cross-population genetic associations, with striking frequency differences between patients and healthy controls. Specifically, HLA-A*33:03 and HLA-C*03:02 each occurred in 10.0% of allopurinol-induced SJS/TEN patients and 28.6% of SCARs patients; the same study also identified HLA-C*08:01 as an additional susceptibility allele with an identical frequency profile. All three alleles were absent in the Italian general population (0.0%). Furthermore, extended haplotypes (B*58:01-DRB1*13:02, DRB1*15:02-DQB1*05:02) remained significantly associated with disease risk. In line with Han Chinese findings (Hung et al., 2005), HLA-A*33:03 and HLA-C*03:02 are in strong linkage disequilibrium with HLA-B*58:01, forming an extended high-risk haplotype that drives ethnic disparities in adverse reaction susceptibility.

##### 2.3.3.2 Protective alleles

In contrast to the aforementioned susceptibility loci, HLA-A*02:01 is the only HLA allele identified to date that confers protection against allopurinol-induced SCARs. This protective effect was rigorously validated in South Korean populations, where HLA-A*02:01 was present in 29.8% of allopurinol-tolerant individuals but completely absent in patients with allopurinol-induced SJS/TEN and SCARs; its frequency in the general South Korean population was 29.9% (Kang et al., 2011). All summarized data regarding HLA allele associations are presented in Table 4.

#### 2.3.4 Non-HLA factors associated with allopurinol-induced SJS/TEN

Beyond HLA genes, additional factors may modulate individual susceptibility to allopurinol-induced SCARs, including renal insufficiency (Hung et al., 2005; Arellano and Sacristán, 1993), viral infections (Suzuki et al., 1998), polymorphisms in drug metabolism genes (e.g., CYP2C9, NAT2), and immune regulatory genes (e.g., IL-15, TNF). However, further research is required to fully elucidate the specific contributions of these factors.

### 2.4 Non-steroidal anti-inflammatory drugs (NSAIDs)

The widespread use of NSAIDs has been linked to a spectrum of adverse drug reactions, varying from mild local manifestations such as skin rashes and gastrointestinal irritation to severe systemic effects and life-threatening SJS/TEN (Imatoh and Saito, 2021). Importantly, concurrent infection can further increase the risk of NSAID-associated SJS/TEN (Imatoh and Saito, 2021). While ibuprofen, naproxen, and aspirin are the most frequently implicated NSAIDs in SJS/TEN cases (Blumenthal et al., 2015), sporadic case reports have also associated other agents including nabumetone with such SCARs (Cristallo et al., 2011). This section addresses the genetic basis, risk profiles of individual agents, and preventive strategies.

#### 2.4.1 Oxicam

A European study has linked oxicam-related TEN cases to HLA-A*02 and HLA-B*12, providing early evidence of genetic susceptibility to NSAIDs (Roujeau et al., 1987). Building on this finding, oxicam-type NSAIDs—including meloxicam, piroxicam, and tenoxicam—are now widely recognized as high-risk agents for SJS/TEN. Subsequent investigations have identified the HLA-B*73:01 allele as a potential key genetic susceptibility factor for this class of drugs (Lonjou et al., 2008). Nevertheless, relevant studies on NSAID-associated genetic susceptibility remain scarce.

#### 2.4.2 Paracetamol

Paracetamol and ibuprofen are frequently used in pediatric populations, and they have been found to be significantly more strongly associated with TEN in younger children (0–11 years old) than in older children (12–17 years old), with a notable male predominance (Bayram-Ozgur et al., 2025). In an Italian study of 20 SJS/TEN cases, 10 were attributed to paracetamol-induced reactions (Cristallo et al., 2011). However, no significant associations were identified between paracetamol-induced SJS/TEN and specific HLA alleles—even a modest increase in the frequency of DQB1*02:02 in some patients lost statistical significance after multiple testing correction (Cristallo et al., 2011). This stands in contrast to allopurinol-induced SJS/TEN, which exhibits well-established HLA and haplotype associations, suggesting that paracetamol-induced cases may be driven by non-HLA genetic factors or environmental triggers rather than stable HLA polymorphisms. Consequently, single HLA genotyping is ineffective for risk stratification of paracetamol-induced SJS/TEN, necessitating a comprehensive multi-factorial assessment that integrates clinical characteristics, exposure history, and potential confounding factors (Cristallo et al., 2011).

#### 2.4.3 Valdecoxib

Valdecoxib was withdrawn from the market in 2005 due to concerns over severe adverse reactions, particularly SJS/TEN. A signal mining study of FAERS by Castellana et al. (2025) identified 1339 cases of valdecoxib-associated SJS/TEN, accounting for 3.40% of all SJS/TEN cases included in the study. Notably, when normalized to the total number of valdecoxib-related adverse event reports, this translates to the highest relative risk (10.71%) among comparable medications. These findings are consistent with research by Fei et al. (2023) , who reported a similar relative risk of 14.99% for valdecoxib-induced SJS/TEN.

#### 2.4.4 Celecoxib

Celecoxib has been identified as one of the key drugs associated with SJS/TEN in the United States (Fei et al., 2023). Among the top 50 drugs linked to SJS/TEN, 71.81% of celecoxib-associated cases were reported in the United States population (Fei et al., 2023). This data underscores the need for heightened clinical vigilance when prescribing celecoxib, particularly in the United States, and emphasizes the importance of careful monitoring for early signs of SJS/TEN (e.g., skin rashes, mucosal lesions) following treatment initiation (Fei et al., 2023).

#### 2.4.5 Diclofenac

Diclofenac was among the top 40 drugs linked to drug-induced SJS/TEN in a FAERS analysis (Mukherjee et al., 2025a). In one reported case, diclofenac suppositories were prescribed for pain management, and the patient’s condition deteriorated after 2 days, leading to a diagnosis of SJS with diclofenac suspected as the culprit (Fa et al., 2024). However, meropenem and metronidazole were also administered during the intensive care unit (ICU) admission (Fa et al., 2024). Metronidazole—a potential high-risk antibiotic for SJS/TEN—typically has a longer latency period compared to NSAIDs, which often induce SJS/TEN within 4–5 days of exposure (Mukherjee et al., 2025a). Notably, some NSAIDs may trigger rashes and mucosal erosion as early as 1–3 days after administration (Monographs, 2025). This temporal overlap raises the possibility that NSAIDs prescribed to alleviate symptoms during the flu-like prodromal phase of SJS/TEN may be mistakenly identified as the causative agent. Additionally, diclofenac should be used with extreme caution in patients with lupus, as it may exacerbate underlying skin conditions and increase the risk of SJS/TEN.

#### 2.4.6 Risk assessment and monitoring for NSAID-induced SJS/TEN

Although the absolute risk of SJS/TEN associated with NSAID use remains low, clinicians should maintain a high index of suspicion when monitoring patients who have recently initiated NSAID therapy (Mockenhaupt et al., 2003)—especially those with concurrent infections, a history of autoimmune diseases (e.g., lupus), or prior adverse cutaneous reactions to medications. NSAIDs may act synergistically to induce or exacerbate SJS/TEN during its critical onset phase, and timely recognition of early symptoms is crucial for reducing morbidity and mortality. For patients at potential high risk (e.g., those with a history of NSAID-induced hypersensitivity), skin patch tests have shown promise for identifying latent susceptibility to NSAID-induced SJS/TEN (Kowalski and Makowska, 2015), offering a potential tool for personalized risk assessment and treatment planning. In addition, when SJS/TEN develops, risks from concomitant potential high-risk medications should also be considered. Given the broad clinical utility of NSAIDs and their widespread use across diverse patient populations, further research is warranted to elucidate the underlying mechanisms of NSAID-induced SJS/TEN, identify additional genetic or environmental risk factors.

### 2.5 Proton pump inhibitors (PPIs)

Emerging evidence indicates an elevated risk of SJS/TEN in patients receiving PPIs. As first-line medications for acid-related disorders (e.g., gastroesophageal reflux disease, peptic ulcers), PPIs—including omeprazole, lansoprazole, rabeprazole, and pantoprazole—have been implicated in SJS/TEN development (Frey et al., 2019; Mockenhaupt et al., 2008). A study of FAERS revealed that PPIs were suspected as the culprit in 11.09% of SJS/TEN cases (Fei et al., 2023). Notably, lansoprazole is the only PPI among the top 50 drugs associated with SJS/TEN, and lansoprazole-associated SJS/TEN is linked to a mortality rate exceeding 50% (Fei et al., 2023)—markedly higher than the overall mortality of SJS/TEN. Regional variations have also been observed: a Taiwanese study (Lin et al., 2018) focusing on PPI-related severe delayed-type hypersensitivity reactions (including SJS/TEN) identified esomeprazole as the most common trigger in this context. These discrepancies may be attributed to differences in prescribing patterns, genetic factors, or population-specific pharmacokinetic profiles.

However, the causal relationship between PPIs and SJS/TEN remains unconfirmed. PPIs are frequently co-administered with other potential high-risk SJS/TEN-inducing medications, which may confound the observed association. Historically, PPIs were not recognized as major triggers of SCARs, and their low immunogenicity further underscores the need for caution when inferring causality from observational data alone. Large-scale, well-designed prospective studies are warranted to clarify the true risk of SJS/TEN associated with PPIs. Future research should adjust for potential confounders (e.g., concurrent medications, comorbidities, genetic background) and stratify analyses by PPI subtype, dosage, and treatment duration. Additionally, mechanistic investigations—such as drug-specific T-cell activation assays and HLA typing studies—may help elucidate the underlying pathophysiological pathways. While accumulating data suggest a potential association between PPIs and SJS/TEN (with variable risks across subtypes and regions), definitive causal evidence is still lacking. Clinicians should maintain vigilance for cutaneous and mucosal reactions in patients receiving PPIs.

### 2.6 Immune checkpoint inhibitors (ICIs)

As the clinical application of ICIs expands beyond advanced malignancies to include early-stage neoplasms and biomarker-driven pan-cancer therapies, the risk of cutaneous adverse reactions has correspondingly increased. These reactions include severe immune-mediated bullous eruptions resembling SJS/TEN, a phenomenon attributed to the enhanced cytotoxicity induced by ICIs (Johnson et al., 2025). Clinically, differentiating ICI-induced “true” SJS/TEN from SJS/TEN-like reactions remains a significant challenge. Notably, the anti-CTLA-4 agent ipilimumab has not been independently associated with SJS/TEN-like reactions (Kuo and Markova, 2022), whereas anti-PD-1/PD-L1 therapies are more frequently linked to SJS/TEN-like reactions than to true SJS/TEN (Molina et al., 2020; Ingen-Housz-Oro et al., 2022). Due to misdiagnosis in 36%–72% of patients at initial presentation (Weinkle et al., 2019; Giraud-Kerleroux et al., 2023), the incidence of SJS/TEN reported in previous studies may have been overestimated.

Numerous studies have confirmed that various ICIs are associated with SJS/TEN or SJS/TEN-like reactions, including pembrolizumab, nivolumab, atezolizumab, cemiplimab (Pathak et al., 2025), adalimumab (Salama and Lawrance, 2009), rituximab (Lowndes et al., 2002), enfortumab vedotin (Nguyen et al., 2021), and others (Kuo and Markova, 2022). Regarding drug-specific risks, nivolumab carries a lower risk compared with other PD-1 inhibitors, while pembrolizumab is associated with the highest mortality rate among related cases (Pathak et al., 2025). Additionally, patients who receive antibiotics during ICI therapy have a significantly higher incidence of SJS/SJS-like reactions than those who do not (Jing et al., 2022); conversely, a study by Mukherjee et al. found a negative association between cancer diagnosis and the risk of SJS/TEN (Mukherjee et al., 2025b). Another study indicated that the initiation of potential high-risk concomitant medications (e.g., TMP-SMX, allopurinol) during ICI therapy in patients without documented drug allergies may be a potential contributing factor to the development of SJS/TEN-like cutaneous toxicities (Molina et al., 2020)—a finding consistent with the interaction analysis by Mukherjee et al., which revealed additive synergy between ICI exposure and culprit medications (Mukherjee et al., 2025b).

SJS/TEN-like reactions typically occur weeks to months after the initiation of ICI treatment (Ellis et al., 2020), but the time to onset (TTO) varies among different agents. An international multicenter study showed that the median TTO of ICI-related SJS/TEN-like reactions was 52 days (interquartile range [IQR]: 3–420) (Oro et al., 2021). A systematic review by Maloney et al. included 12 cases of SJS/TEN-like reactions (12 SJS; 5 TEN) and found that nivolumab (7 cases) had a median TTO of 3 weeks, whereas pembrolizumab (5 cases) exhibited a notably longer median onset time of 11 weeks (Maloney et al., 2020). However, a retrospective analysis of 42 pembrolizumab-induced SJS/TEN cases (20 SJS; 13 TEN; 9 SJS-TEN overlap) reported a median TTO of only 15 days (IQR: 2–180) (Wu et al., 2025). For enfortumab vedotin-associated SJS/TEN, the median TTO was even shorter, at 11 days (IQR: 9–21 days) (Nguyen et al., 2021).

In terms of clinical features and prognosis, SJS/TEN-like reactions are generally less severe than true SJS/TEN and more responsive to treatment (Molina et al., 2020). Maloney et al. observed that SJS-like rashes responded well to systemic therapy and supportive care with no associated deaths, whereas TEN-like reactions were more severe, with 3 out of 5 patients (60%) succumbing (Maloney et al., 2020). Pembrolizumab-related cases had a mortality rate of 14.3%, with 9.5% attributed to SJS/TEN (134). For enfortumab vedotin, the mortality rate reached 50% (4/8) (Nguyen et al., 2021). The median age at onset of pembrolizumab-induced SJS/TEN is 65 years (Wu et al., 2025). For elderly patients with comorbidities—an at-risk subgroup—clinicians should maintain a low threshold for drug discontinuation and promptly initiate aggressive management (Chang et al., 2025).

Notably, the consideration of ICI rechallenge for SJS/TEN-like eruptions stands in contrast to the general recommendation for SJS/TEN induced by other medications, where rechallenge with the causative drug is typically contraindicated due to the high risk of life-threatening recurrent reactions. For ICIs, however, rechallenge may be cautiously pursued in selected patients after complete resolution of cutaneous/extracutaneous involvement (grade ≤1), following a multidisciplinary evaluation of drug-associated eruption severity, concurrent immunosuppressant use, prior cancer response to ICIs, and alternative anti-cancer therapies (Molina et al., 2020; Brahmer et al., 2021). This distinction stems from the unique mechanism of immune checkpoint inhibition and the frequent paucity of alternative treatment options for advanced cancers, requiring a balance between the risk of recurrent SCARs and the potential anti-tumor efficacy of continued ICI therapy. In a study of pembrolizumab-induced SJS/TEN, a total of 4.8% of patients (2 cases) underwent rechallenge and did not develop recurrent SJS/TEN (Wu et al., 2025). However, ICI rechallenge is clearly contraindicated in patients with true SJS/TEN.

### 2.7 Novel antiandrogen agents

To systematically and comprehensively evaluate the safety profile of novel androgen receptor inhibitors (including apalutamide, abiraterone, enzalutamide, darolutamide, etc.), particularly to clarify the association between these agents and SJS/TEN as well as the differences in associated risks, one research team conducted a targeted pharmacovigilance analysis (Zhu et al., 2025). This analysis integrated real-world data from three major global adverse drug reaction (ADR) databases—FAERS, EudraVigilance and JADER—covering the period from Q1 2018 to Q1 2024. Key findings from this analysis uncovered notable lethality associated with apalutamide-related SJS/TEN: The fatality rate of apalutamide-induced SJS is 24.4%, while that of apalutamide-related TEN reaches 51.1%—both substantially higher than the typical rates reported in clinical literature (Traikia et al., 2020)—further underscoring the severe nature of this apalutamide-associated adverse reaction. Regarding the TTO, following apalutamide initiation, the median latency period was 41 days for SJS and 29 days for TEN; this time frame is consistent with the results of a prior study by Katsuta et al., which observed a median TTO of 5.2 weeks (≈36 days) for antiandrogen-associated TEN (138). This consistency provides robust support for the temporal association between TEN and antiandrogen exposure. In stark contrast, no disproportional reporting signal for SJS/TEN was detected among patients receiving abiraterone, enzalutamide, or darolutamide. This discrepancy suggests that potential differences may exist in the immunotoxicity profiles of distinct classes of novel antiandrogens, with heterogeneous risks of inducing severe immune-mediated cutaneous adverse reactions. These findings offer valuable insights for clinical medication selection and safety monitoring.

### 2.8 Carbonic anhydrase inhibitors

Methazolamide (MTZ) is a typical sulfonamide-derived carbonic anhydrase inhibitor primarily indicated for the clinical management of glaucoma. As a sulfonamide-class drug, MTZ carries a risk of SCARs, including SJS/TEN, with a risk profile comparable to other sulfonamide derivatives. Notably, to date, MTZ-induced SJS/TEN has been exclusively documented in individuals of Asian ancestry harboring specific HLA gene mutations (Baluch et al., 2022). Accumulating evidence has firmly linked the HLA-B*59 allele group, particularly the HLA-B*59:01 allele, to MTZ-induced SJS/TEN in Korean, Japanese, and Han Chinese populations. In Korean (Kim et al., 2010) and Japanese (Shirato et al., 1997) cohorts, the carrier rate of HLA-B*59:01 reaches 100% and 75% in MTZ-induced SJS/TEN cases, respectively, in stark contrast to the low background frequencies of 4.1% and 3.8% in the corresponding general populations. Similarly, among Han Chinese individuals diagnosed with MTZ-induced SJS/TEN, the HLA-B*59:01 allele carrier rate ranges from 66.7% to 87.5%, while the allele frequency is merely 0.4% in the Chinese general population and 0.0% in MTZ-tolerant control subjects, further consolidating the high specificity of this genetic biomarker for predicting MTZ-induced SJS/TEN (Yang et al., 2016).

Beyond the well-characterized association with HLA-B*59:01 (Shu et al., 2015), recent research has identified HLA-B*55:02 as a novel risk allele for MTZ-induced SJS/TEN in Han Chinese individuals (Jiang et al., 2022). Specifically, 66.7% of MTZ-induced SJS/TEN patients who tested negative for HLA-B*59:01 were found to carry the HLA-B*55:02 allele, in sharp contrast to only 2.7% of MTZ-tolerant controls; the overall prevalence of HLA-B*55:02 in MTZ-induced SJS/TEN patients reaches 22.2% (Jiang et al., 2022). Mechanistically, the E45-L116 amino acid motif within the HLA-B protein has been shown to fully explain the pathogenic association of both HLA-B*59:01 and HLA-B*55:02 with MTZ-induced SJS/TEN (Jiang et al., 2022). Furthermore, the rs41562914(A)-rs12697944(A) haplotype, which encodes this critical E45-L116 variant, has emerged as a robust genetic predictor for this severe adverse reaction (Jiang et al., 2022).

In Korean patients with MTZ-induced SJS/TEN, HLA-C*01:02 and HLA-A*24:02 were significantly more prevalent than in the general population, in addition to the established risk allele HLA-B*59:01 (Kim et al., 2010). Specifically, HLA-C*01:02 was detected in 100% of Korean MTZ-induced SJS/TEN patients, whereas its frequency in the general population was 33.2%. Similarly, HLA-A*24:02 was present in 80.0% of SJS/TEN cases, in contrast to 38.8% in the general cohort. In Han Chinese populations, HLA-C*01:02 was reported to be in strong linkage disequilibrium with HLA-B*59:01 (Yang et al., 2016). Although its independent association with MTZ-induced SJS/TEN is comparatively weaker than that of HLA-B*59:01 or HLA-B*55:02, the consistent presence of HLA-C*01:02 in high-risk genetic backgrounds reinforces its value as a secondary, yet notable, genetic risk modifier in this population (Yang et al., 2016).

The above-mentioned data are detailed in Table 5. Given the consistent identification of HLA-related genetic risk factors across multiple Asian ethnic groups, pre-treatment genetic screening for these specific HLA alleles is strongly warranted in Asian populations prior to the initiation of MTZ therapy (Yang et al., 2016; Tangamornsuksan and Lohitnavy, 2019). Such proactive screening strategies hold significant promise for mitigating the risk of SJS/TEN and improving the overall safety of MTZ use in high-risk patient cohorts.

**TABLE 5:** Distribution of key HLA alleles across MTZ-related populations: SJS/TEN, SCARs, tolerant individuals, and general population.

HLA	Population	SJS/TEN	SCARs	Tolerant	General
HLA-B*59:01	Korean (Kim et al., 2010)	100.0% (5/5)	-	-	4.1% (20/485)
	Japanese (Shirato et al., 1997)	75.0% (3/4)	-	-	3.8% (14/371) (Lee et al., 2005; Kim et al., 2010)
	Han Chinese (Yang et al., 2016; Jiang et al., 2022)	66.7% (12/18)-87.5% (7/8)	-	0.0% (0/30)-1.4% (1/74)	0.4% (1/283)
HLA-B*55:02	Han Chinese (Jiang et al., 2022)	22.2% (4/18)	-	2.7% (2/74)	-
HLA-C*01:02	Korean (Kim et al., 2010)	100.0% (5/5)	-	-	33.2% (161/485)
HLA-A*24:02	Korean (Kim et al., 2010)	80.0% (4/5)	-	-	38.8% (188/485)

SJS/TEN, Stevens-Johnson syndrome/toxic epidermal necrolysis; HLA, human leukocyte antigen; MTZ, methazolamide; SCARs, severe cutaneous adverse reactions; Tolerant = tolerant population exposed to the relevant trigger but without SJS/TEN, onset; General = unselected general population; “-” = missing data due to no relevant literature reports.

### 2.9 Antivirals

Antiviral medications are widely utilized for the management of various viral infections, including human immunodeficiency virus (HIV) and herpesvirus diseases. Although generally well-tolerated, several antiviral agents have been implicated in the development of life-threatening SJS/TEN, with distinct risk profiles observed across different drug classes. Below, we address abacavir hypersensitivity syndrome (ABC-HSR), and examine the risk of SJS/TEN associated with nevirapine, other antiretrovirals, valaciclovir, and acyclovir.

#### 2.9.1 ABC-HSR

The association between HLA-B*57:01 and abacavir hypersensitivity—among which SJS/TEN represents a severe manifestation—pioneered the field of ADR-HLA genotype correlations and remains a mandatory genetic screening requirement per drug labeling. Notably, HLA-B*57:01 has a higher allele frequency in Caucasian populations: its prevalence is highest in white populations (6.49%) and lowest in black populations (0.39%), a difference that leads to a substantially higher incidence of ABC-HSR in Caucasians than in non-Caucasian groups (Orkin et al., 2010; To et al., 2013). This allele demonstrates 100% sensitivity in predicting immunologically confirmed ABC-HSR, and the corresponding genetic test boasts the lowest Number Needed to Genotype (NNG) among similar HLA-based screening tools for drug-induced SCARs such as SJS/TEN. It is followed in clinical utility by HLA-B*58:01 screening for allopurinol-induced SCARs and HLA-B*15:02 screening for CBZ-induced SJS/TEN (Manson et al., 2020). A double-blind, prospective, multicenter randomized study (Mallal et al., 2008) showed that the incidence of ABC-HSR was significantly lower in patients who received prospective HLA-B*57:01 screening (3.4%, 27/803) than in those in the unscreened control group (7.8%, 66/847). Meanwhile, the median time from abacavir initiation to symptom onset was 10 days (IQR: 3–14) in the screened group, versus 9 days (IQR: 5–12) in the control group. Beyond HLA-B*57:01, HLA-DRB1*07 and HLA-DQB1*03 are also key susceptibility genetic markers for abacavir hypersensitivity; each alone or in combination is significantly associated with an increased risk of this hypersensitivity (Mallal et al., 2002). Specifically, in this western Australian study cohort, the combined presence of the three markers shows a 100% positive predictive value and 97% negative predictive value for ABC-HSR (Mallal et al., 2002).

#### 2.9.2 Nevirapine and other antiretroviral agent-induced SJS/TEN

Pinpointing the specific antiretroviral agent responsible for drug eruptions in HIV-infected patients is often challenging, as these medications are rarely administered as monotherapy. Nevirapine (NVP), a non-nucleoside reverse transcriptase inhibitor (NNRTI) used for HIV treatment, carries substantial cutaneous toxicity risks—with SJS listed as a severe ADR under a boxed warning in its labeling. Although implementing a 2-week lead-in phase with 200 mg daily, followed by a maintenance regimen of 200 mg twice daily, may reduce the overall risk of rash, SJS can still occur (Warren et al., 1998; Metry et al., 2001). Nevirapine is significantly associated with an increased risk of SJS/TEN in HIV-infected patients, with a median TTO of 12 days (IQR: 10–240) following administration, even with adherence to lead-in or dose-escalation protocols (Fagot et al., 2001). Immediate discontinuation of nevirapine is strongly recommended upon the appearance of any cutaneous eruption. In a Mozambican population, the CYP2B6 single nucleotide polymorphisms (SNPs) G516T and T983C have been associated with susceptibility to NVP-induced SJS/TEN: notably, the 983C allele confers a significantly heightened risk of developing SJS/TEN (with homozygous status exclusively observed in cases), while the GT haplotype (wildtype for both SNPs) exerts a statistically significant protective effect (Ciccacci et al., 2013). As SJS/TEN is a complex disorder involving multiple genetic and non-genetic factors, CYP2B6 represents only one of several contributing elements. Further research is imperative to explore the potential role of the HLA system in NVP-induced SJS/TEN—with the goal of integrating findings on CYP2B6 genetic variability with the immune system’s involvement—while considering this metabolic enzyme gene polymorphism in risk assessment for NVP-related SCARs. Beyond nevirapine, the nucleoside reverse transcriptase inhibitors (NRTIs) zidovudine (Murri et al., 1996) and didanosine (Parneix-Spake et al., 1992), as well as the protease inhibitor indinavir (Teira et al., 1998), have also been documented to induce SJS/TEN.

#### 2.9.3 Acyclovir and valaciclovir-induced SJS/TEN

A signal mining analysis of SCARs using the FAERS database confirmed positive signals for SJS/TEN linked to both valaciclovir and acyclovir (Mao et al., 2024). While the incidence of these SCARs remains relatively low, 58 SJS/TEN cases have been documented in association with valaciclovir and 117 cases with acyclovir—findings that underscore non-negligible risks for both antiviral agents. Distinct differences in onset time were observed between the two drugs: for TEN, the median TTO was 17 days in valaciclovir-treated patients versus 12 days in those receiving acyclovir; for SJS, the median TTO was 12 days for valaciclovir compared to 8 days for acyclovir. These data suggest that acyclovir-induced SJS/TEN may manifest slightly earlier than valaciclovir-associated cases. Regarding age-related distribution, the proportion of pediatric patients (<18 years old) affected by SJS and TEN was higher than that of other SCARs subtypes such as drug reaction with eosinophilia and systemic symptoms (DRESS) and acute generalized exanthematous pustulosis (AGEP), highlighting that pediatric populations may be more susceptible to SJS/TEN following exposure to these two antiviral drugs. Notably, the potential risks of SJS/TEN have been formally incorporated into acyclovir’s drug label, reflecting clinical recognition and emphasis on these severe ADRs. Collectively, these findings underscore the need for vigilant monitoring of skin and mucosal manifestations during treatment with valaciclovir or acyclovir—particularly in pediatric patients—and prompt drug discontinuation if suspicious symptoms emerge, to mitigate the progression of severe reactions.

### 2.10 Miscellaneous drugs

In addition to the aforementioned medications, a broad spectrum of other pharmaceutical agents has been documented as potential triggers of SJS/TEN, including the following classes and their representative drugs: antidepressants such as fluoxetine (Frey et al., 2019), mirtazapine (Frey et al., 2019), sertraline (Mukherjee et al., 2025a), and bupropion (Blumenthal et al., 2015); antihypertensives covering selective calcium channel blockers such as amlodipine (Fei et al., 2023), beta-blockers (Blumenthal et al., 2015), and angiotensin-converting enzyme inhibitors (ACEIs) (Blumenthal et al., 2015); antituberculosis drugs (Morán-Mariños et al., 2025); anticoagulants including enoxaparin (Mukherjee et al., 2025a); immunosuppressants including lenalidomide (Fei et al., 2023; Alleg et al., 2012); antifungal agents including fluconazole (Mukherjee et al., 2025a), terbinafine (Banik et al., 2021) and itraconazole (Cristallo et al., 2011); synthetic glucocorticoids including deflazacort (Li et al., 2024b); muscle relaxants including chlormezanone (Mockenhaupt et al., 2008); diuretic drug including furosemide (Castellana et al., 2025; Fei et al., 2023); antineoplastic agents such as sorafenib (Mukherjee et al., 2025a), methotrexate (Fei et al., 2023), tamoxifen (Madabhavi et al., 2015), vemurafenib (Bellón et al., 2016), pemetrexed (Eichhoff, 2020), and carboplatin (Eichhoff, 2020); antirheumatic drugs including hydroxychloroquine (Fei et al., 2023; Blumenthal et al., 2015); and 5-aminosalicylates (Frey et al., 2019) encompassing sulfasalazine and mesalamine. The association between these drugs and SJS/TEN remains weak and poorly established. Further rigorous epidemiological surveys, pharmacovigilance validation, and mechanistic research are warranted to verify a potential causal link.

## 3 SJS/TEN and related hypersensitivity reactions onset time variability

Comprehensive analysis of the TTO of SJS/TEN induced by distinct drug classes demonstrates substantial heterogeneity, which is strongly correlated with drug characteristics and underlying pharmacological mechanisms. TTO data for SJS/TEN and associated hypersensitivity reactions derived from multiple sources are summarized in Table 6. The overall median TTO among all causative medications was 12.0 days (Mukherjee et al., 2025a).

**TABLE 6:** Median onset time variability across SJS/TEN and related hypersensitivity reactions.

Drug class	Median onset time
Overall	Overall medications (Mukherjee et al., 2025a): 12.0 days
Antibiotics	Overall antibiotics (Yan et al., 2025): 4.5 days for SJS; 7.5 days for TEN Oxazolidinones-Linezolid (Ni et al., 2022): 5.0 days Carbapenems**: 6.0 days (Fukasawa et al., 2023) Overall penicillins (including β-lactamase inhibitors)**: 7.0 days (Fukasawa et al., 2023); Amoxicillin* (Mukherjee et al., 2025a): 6.0 days; Clavulanate* (Mukherjee et al., 2025a): 6.0 days Overall cephalosporins (including β-lactamase inhibitors)**: 13.0 days (Fukasawa et al., 2023); Cefotaxime: 32.5 days (Yan et al., 2025); Ceftriaxone* (Mukherjee et al., 2025a): 4.0 days Overall macrolides**: 7.0 days (Fukasawa et al., 2023); Clarithromycin* (Mukherjee et al., 2025a): 7.0 days; Azithromycin* (Mukherjee et al., 2025a): 4.0 days Overall lincomycins**: 7.0 days (Fukasawa et al., 2023); Clindamycin* (Mukherjee et al., 2025a): 5.0 days Overall tetracyclines**: 11.0 days (Fukasawa et al., 2023) Overall quinolones**: 11.0 days (Fukasawa et al., 2023); Levofloxacin* (Mukherjee et al., 2025a): 6.0 days; Ciprofloxacin* (Mukherjee et al., 2025a): 7.0 days Overall glycopeptides**:16.5 days (Fukasawa et al., 2023); Vancomycin:12.0 days for SJS (31)/12.0 days* (Mukherjee et al., 2025a)/12.5 days for SJS (17) TMP-SMX**: 17.0 days (Fukasawa et al., 2023); TMP/SMX Inj* (Mukherjee et al., 2025a): 7.0 days Diaminopyrimidines: Trimethoprim* (Mukherjee et al., 2025a): 9.0 days
Antiepileptics * (Mukherjee et al., 2025a)	Levetiracetam: 10.0 days; Carbamazepine: 13.0 days; Oxcarbazepine: 17.0 days; Lamotrigine: 17.0 days; Zonisamide: 19.0 days; Phenytoin: 23.0 days
Xanthine oxidase inhibitors	Allopurinol* (Mukherjee et al., 2025a): 20.0 days
NSAIDs	Overall NSAIDs: 4.0–5.0 days (Mukherjee et al., 2025a) Ibuprofen* (Mukherjee et al., 2025a): 4.0 days; Acetaminophen* (Mukherjee et al., 2025a): 5.0 days; Diclofenac* (Mukherjee et al., 2025a): 6.0 days; Valdecoxib* (Mukherjee et al., 2025a): 9.0 days; Aspirin* (Mukherjee et al., 2025a): 17.0 days
PPIs* (Mukherjee et al., 2025a)	Omeprazole: 11.0 days; Lansoprazole: 14.0 days; Esomeprazole: 14.0 days; Pantoprazole: 22.0 days
ICIs	Overall ICIs SJS/TEN-like reactions (Oro et al., 2021): 52.0 days Enfortumab vedotin (Nguyen et al., 2021):11.0 days Nivolumab: 3.0 weeks (Maloney et al., 2020)/21.0 days* (Mukherjee et al., 2025a) Pembrolizumab:15.0 days (Wu et al., 2025)/19.0 days* (Mukherjee et al., 2025a)/11.0 weeks (Maloney et al., 2020)
Novel antiandrogens	Overall Antiandrogen (Katsuta et al., 2024): 36.0 days for TEN Apalutamide (Zhu et al., 2025): 41.0 days for SJS; 29.0 days for TEN
Antivirals	ABC-HSR(148): 9.0 days for HLA-B*57:01 unscreened; 10.0 days for HLA-B*57:01 screened Nevirapine:12.0 days (Fagot et al., 2001)/18.0 days* (Mukherjee et al., 2025a) Valaciclovir (Mao et al., 2024): 12.0 days for SJS; 17.0 days for TEN Acyclovir (Mao et al., 2024): 8.0 days for SJS; 12.0 days for TEN
Non-ICI antineoplastic agents* (Mukherjee et al., 2025a)	Sorafenib: 9.0 days; Carboplatin: 11.0 days; Enfortumab: 14.0 days; Lenalidomide: 16.0 days
Others* (Mukherjee et al., 2025a)	Fluconazole: 10.0 days; Furosemide: 11.0 days; Sertraline: 14.0 days; Enoxaparin: 16.0 days; Sulfasalazine: 17.0 days; Amlodipine: 18.0 days

SJS/TEN, Stevens-Johnson syndrome/toxic epidermal necrolysis; TMP/SMX, Inj = trimethoprim-sulfamethoxazole injection; NSAIDs, nonsteroidal anti-inflammatory drugs; PPIs = proton pump inhibitors; ICIs, immune checkpoint inhibitors; ABC-HSR = abacavir hypersensitivity syndrome; “*” = Data on TTO, were obtained from figures in a FAERS, data mining study of the top 40 drugs most frequently associated with SJS/TEN., as the data were derived from graphical extraction, the reported decimal places are approximate rather than precise values. “**” = Data on TTO, were derived from SJS/TEN, cases assessed by the ALDEN, scoring system.

### 3.1 Medications with short median TTO for SJS/TEN

Antibiotics generally exhibit a short latency profile. The median TTO for antibiotic-associated SJS and TEN was 4.5 days (IQR: 1.5–10.75) and 7.5 days (IQR: 2.5–16.5), respectively, indicating a significantly later onset of TEN compared with SJS (Yan et al., 2025). Most antibiotics demonstrate short TTO, such as ceftriaxone (4.0 days) (Mukherjee et al., 2025a), azithromycin (4.0 days) (Mukherjee et al., 2025a), and linezolid (5.0 days) (Ni et al., 2022). Detailed TTO values for other medications are presented in Table 6. Notably, prolonged TTO was seen with TMP-SMX (17.0 days; IQR: 12.0–48.0) (Fukasawa et al., 2023) and cefotaxime, which showed the longest latency at 32.5 days (Yan et al., 2025). Intriguingly, the median TTO of intravenous TMP-SMX was merely 7.0 days (Mukherjee et al., 2025a). This discrepancy can be mechanistically explained by the rapid achievement of high concentrations of sensitizing metabolites after intravenous administration, the lack of first-pass metabolism resulting in direct systemic immune exposure, potential hypersensitivity to intravenous excipients that may accelerate the onset of SJS/TEN, and the rapid reactivation of memory T cells in pre-sensitized individuals, rather than differences in the pharmacokinetics of the parent drug alone. Vancomycin was associated with a median TTO of 12.5 days (Yan et al., 2025), consistent with two other studies reporting 12.0 days (Mukherjee et al., 2025a; Ni et al., 2022). However, among SJS/TEN cases defined by the ALDEN scoring system, the median TTO for glycopeptides (including vancomycin) was 16.5 days (IQR: 9.0–24.0). This discrepancy may be partly due to stricter case ascertainment and more reliable onset time documentation under ALDEN-based case definition. Other medication-specific TTO values for SJS/TEN defined by the ALDEN scoring system were as follows (Fukasawa et al., 2023): carbapenems, 6.0 days (IQR: 6.0–6.0); penicillins including β-lactamase inhibitors, 7.0 days (IQR: 5.0–9.0); macrolides, 7.0 days (IQR: 4.0–8.0); lincomycins, 7.0 days (IQR: 7.0–7.0); tetracyclines, 11.0 days (IQR: 11.0–11.0); quinolones, 11.0 days (IQR: 7.0–12.0); and cephalosporins including β-lactamase inhibitors, 13.0 days (IQR: 7.0–22.0).

Similarly, NSAIDs also exhibit an overall short latency, with a median TTO range of 4.0–5.0 days (Mukherjee et al., 2025a). The majority of NSAIDs have a short TTO, such as ibuprofen (4.0 days), acetaminophen (5.0 days), and diclofenac (6.0 days), while prolonged TTO is observed for select agents, including valdecoxib (9.0 days) and aspirin (17.0 days).

Both antibiotics and NSAIDs are associated with rapid symptom onset, typically occurring within days of drug administration. This short TTO profile can be attributed to rapid haptenization, direct immune stimulation, and direct pharmacological interactions with immune receptors that trigger swift immune activation. It should be noted that short TTO cases are more likely to be reported, whereas long-latency cases are prone to under recognition, leading to the apparent infrequency of prolonged TTO. Furthermore, the risk of misclassifying individual agents as the definitive causative drug within these two classes cannot be excluded.

### 3.2 Medications with moderate median TTO for SJS/TEN

Most other drugs associated with SJS/TEN fall into the moderate latency category, with a median TTO ranging from 10.0 to 23.0 days. This intermediate latency profile is largely attributed to slow metabolic bioactivation, time-dependent clonal expansion of drug-specific T cells, and gradual tissue accumulation of immunogenic agents, all of which contribute to a delayed yet robust immune response. This broad category encompasses several classes of medications: individual agents such as allopurinol with a TTO of 20.0 days (Mukherjee et al., 2025a), the IQR of TTO was 14.0–34.0 days (Tho et al., 2002); antiepileptic drugs including levetiracetam (10.0 days), CBZ (13.0 days), OXC (17.0 days), LTG (17.0 days), zonisamide (19.0 days), and phenytoin (23.0 days); and PPIs consisting of omeprazole (11.0 days), lansoprazole (14.0 days), esomeprazole (14.0 days), and pantoprazole (22.0 days) (Mukherjee et al., 2025a). Among antiviral agents, ABC-HSR has a TTO of 9.0–10.0 days (Mallal et al., 2008), nevirapine demonstrates variable TTO values across studies (12.0 days (Fagot et al., 2001) and 18.0 days (Mukherjee et al., 2025a)), valaciclovir shows a TTO of 12.0 days for SJS and 17.0 days for TEN, and acyclovir exhibits a TTO of 8.0 days for SJS and 12.0 days for TEN (157). Additionally, non-ICI antineoplastic agents in this category include sorafenib (9.0 days), carboplatin (11.0 days), enfortumab (14.0 days), and lenalidomide (16.0 days). A range of other miscellaneous drugs also belong to this latency group, namely, fluconazole (10.0 days), furosemide (11.0 days), sertraline (14.0 days), enoxaparin (16.0 days), sulfasalazine (17.0 days), and amlodipine (18.0 days) (Mukherjee et al., 2025a).

### 3.3 Medications with prolonged median TTO for SJS/TEN

A subset of drugs demonstrates markedly prolonged latency approaching 1 month or longer, such as antiandrogens (36.0 days for TEN) (Katsuta et al., 2024) and apalutamide (41.0 days for SJS; 29.0 days for TEN) (Zhu et al., 2025). This protracted onset primarily stems from slow clonal expansion of autoreactive T cells, non-immediate disruption of immune homeostasis, and superimposed tissue accumulation coupled with chronic inflammation: unlike rapid immune activation triggered by small-molecule agents, ICIs induce sustained immune dysregulation rather than direct immune stimulation, whereby the reversal of immunosuppressive signaling drives gradual proliferation and tissue infiltration of autoreactive T cells, and the comprehensive remodeling of the immune network requires an extended timeframe to manifest severe cutaneous toxicity. Accordingly, ICIs present the longest onset time: the median TTO for ICIs associated with SJS/TEN-like reactions is 52.0 days (Oro et al., 2021), with highly variable onset (some cases delayed for months after treatment initiation) and inconsistent latency reports for the same agent across different studies (Nguyen et al., 2021; Oro et al., 2021; Maloney et al., 2020; Wu et al., 2025; Katsuta et al., 2024). Further large-scale prospective studies and real-world pharmacovigilance data are warranted to clarify the latency distribution of individual ICIs, identify risk modifiers for delayed onset, and elucidate the heterogeneous immune pathogenesis underlying this extreme TTO variability.

### 3.4 Patient-specific factors for SJS/TEN TTO

Beyond drug-specific immunological and pharmacological mechanisms, latency disparities in SJS/TEN are further modulated by key patient-specific factors, as emphasized by (Mukherjee et al., 2025a), who pioneered the application of random survival forests (RSFs) for SJS/TEN pharmacovigilance analysis. These modulators include age, sex, geographic regional variations (stemming from differences in diagnostic criteria and adverse reaction surveillance), and the number of concomitant medications administered. Notably, younger patients consistently exhibit shorter TTO even after adjusting for the type of causative drug, a phenomenon potentially linked to more efficient antigen processing capabilities and higher metabolic turnover rates in this population. In contrast, elderly patients are more prone to polypharmacy and frequent concomitant medication use, which elevates their overall susceptibility to SJS/TEN and further shapes the observed latency profiles.

In summary, the distinct class-specific variations in SJS/TEN TTO arise from the synergistic interplay between drug-specific pharmacological properties and unique individual patient characteristics. Moreover, spontaneous reports derived from database mining are subject to reporting bias and inaccurate timing, thus only serving as a reference. Importantly, TTO is significantly shorter in recurrent SJS/TEN following previous episodes, with rechallenge reactions often occurring within hours to days (Zimmerman and Dang, 2019); thus, re-exposure to the causative drug must be strictly avoided.

## 4 Drug-specific mechanisms and shared immunological pathways in SJS/TEN

Previous mechanistic studies on SJS/TEN (Hasegawa and Abe, 2024; Hasegawa and Abe, 2020; Kocaaga and Kocaaga, 2025; Hsu et al., 2021) have gradually established a unified framework centered on “drug-specific initiation and shared pathway execution,” laying a core theoretical foundation for deciphering the pathogenesis of these diseases. At the level of drug-specific mechanisms, disease initiation hinges on the specific interplay between causative agents, genetic background, and immune recognition molecules: diverse culprit drugs form complexes with distinct HLA alleles (e.g., CBZ-HLA-B*15:02, allopurinol-HLA-B*58:01, abacavir-HLA-B*57:01) through the hapten/pro-hapten model, pharmacological interaction (p-i) concept, or altered peptide model (Pichler, 2002; Adam et al., 2011; Abe, 2015). This interaction subsequently activates drug-specific CD8^+^ T cells with restricted T-cell receptor (TCR) clonotypes, and this drug-HLA-TCR mediated initiation process exhibits significant ethnic disparities—consistent with the ethnic differences in drug-HLA associations detailed earlier (Friedmann et al., 2003). At the level of shared immunological pathways, irrespective of the causative drug, the effector phase of SJS/TEN adheres to a unified immune damage cascade: CD8^+^ cytotoxic T cells serve as the core effector cells, inducing extensive keratinocyte death via multiple mechanisms, including Fas/Fas ligand (FasL)-mediated apoptosis, perforin/granzyme B-triggered caspase cascade activation, granulysin-mediated direct cytotoxicity, and Annexin A1-formyl peptide receptor 1 (FPR1) axis-induced necroptosis (Elmore, 2007; Viard et al., 1998; Saito et al., 2012; Abe et al., 2009; Saito et al., 2014). Concurrently, pro-inflammatory cytokines such as tumor necrosis factor-α (TNF-α), interferon-γ (IFN-γ), and interleukin-15 (IL-15) form an inflammatory amplification network, which further aggravates epidermal damage and tissue destruction. Integrating mechanistic insights with the present pharmacovigilance results reveals consistent biological plausibility across drug classes. Drugs with well-defined HLA restrictions, specific immune-activating properties, and established pathogenic mechanisms (e.g., CBZ, allopurinol, TMP-SMX, abacavir) show strong and consistent signals in surveillance data, supporting their definitive causal association with SJS/TEN. In contrast, many other agents with weak or inconsistent epidemiological signals, including various non-sulfonamide antibiotics, most common NSAIDs, PPIs, ICIs and other miscellaneous drugs, lack defined HLA associations, demonstrate unclear pharmacological immune activation, and have limited mechanistic evidence. Such divergence aligns precisely with the “drug-specific initiation” paradigm: drugs without defined molecular triggers cannot generate robust, reproducible pharmacovigilance signals. This integrative view therefore reinforces that real-world safety signals and mechanistic evidence are mutually supportive and biologically consistent. Collectively, these findings underscore that a unified exploration of the shared and drug-specific immunological pathways underlying SJS/TEN not only clarifies the core principle that “specific initiation dictates disease susceptibility while shared pathways determine the severity of tissue damage” but also provides crucial theoretical support for disease risk screening (e.g., HLA genotyping), early diagnosis (e.g., application of biomarkers such as granulysin and receptor-interacting protein kinase 3 [RIP3]), and targeted therapy (e.g., TNF-α antagonists and cyclosporine intervention). This highlights substantial conceptual value and profound clinical translational significance.

## 5 Limitations

ADR database mining has important limitations. Spontaneous reporting systems are plagued by underreporting, duplication, incomplete data, and reporting bias, all of which compromise data quality and reliability (Yang et al., 2025b). Without accurate denominator data for drug-exposed populations, these databases can only detect exploratory, hypothesis-generating adverse event signals rather than calculate true incidence rates or establish definitive causality between drugs and SJS/TEN. Notably, the potential high-risk drugs identified in this review through disproportionality analyses represent preliminary signals whose associations with SJS/TEN require further validation, particularly for drugs lacking known susceptibility genetic markers. For drugs with available genetic evidence, associations supported by multiple replicated studies and well-characterized HLA alleles carry greater clinical certainty. In contrast, markers identified only in small or single studies are weakly validated and require further independent replication. Moreover, observed genetic associations may be confounded by linkage disequilibrium, and many signals remain population-specific without broad clinical endorsement or guideline support.

## 6 Conclusion

This review summarizes recent advances in SJS/TEN research, focusing on the critical value of ADR database mining in exploring potential drug associations with SJS/TEN. Database analyses are largely consistent with previous reports while identifying several novel candidate high-risk medications, thereby systematically supplementing the existing spectrum of drugs implicated in SJS/TEN. The median TTO of these potential high-risk drugs is also characterized, offering clinically meaningful references for rational medication use and early recognition. Future studies are warranted to validate the suspected associations between these candidate drugs and SJS/TEN. Further research is also needed to clarify the causal links among medications, genetic susceptibility, and SJS/TEN, with the goal of improving the robustness and translational utility of related evidence.
